# Measuring Geographic Inequalities: Dealing with Multiple Health Resources by Data Envelopment Analysis

**DOI:** 10.3389/fpubh.2018.00053

**Published:** 2018-02-28

**Authors:** Martin Dlouhý

**Affiliations:** ^1^Faculty of Informatics and Statistics, University of Economics Prague, Prague, Czech Republic

**Keywords:** geographic inequality, resource allocation, health resources, data envelopment analysis, Czech Republic

## Abstract

The existence of geographic differences in health resources, health expenditures, the utilization of health services, and health outcomes have been documented by a lot of studies from various countries of the world. In a publicly financed health system, equal access is one of the main objectives of the national health policy. That is why inequalities in the geographic allocation of health resources are an important health policy issue. Measures of inequality express the complexity of variation in the observed variable by a single number, and there is a variety of inequality measures available. The objective of this study is to develop a measure of the geographic inequality in the case of multiple health resources. The measure uses data envelopment analysis (DEA), which is a non-parametric method of production function estimation, to transform multiple resources into a single virtual health resource. The study shows that the DEA originally developed for measuring efficiency can be used successfully to measure inequality. For the illustrative purpose, the inequality measure is calculated for the Czech Republic. The values of separate Robin Hood Indexes (RHIs) are 6.64% for physicians and 3.96% for nurses. In the next step, we use combined RHI for both health resources. Its value 5.06% takes into account that the combinations of two health resources serve regional populations.

## Introduction

There is no doubt that in a publicly financed health system, equal access to health services is one of the main objectives of the national health policy (1, 2). The importance of this objective represents an essential element that affects the overall organization of the national health system. Analyzing the geographic distribution of health resources that are necessary for the provision of health services is about measuring variations. The question is whether the observed variations in health resources reflect the variations in the real health needs of the regional population. If it is not the case, then, the resource variations are a sign of health policy failure provided that equal geographic access was stated as a policy objective. The free market allocates health resources according to the willingness and ability to pay, not according to the health needs of the local population. So, a supply of health services will be concentrated in rich areas, whereas poor areas, albeit being usually those with the greatest health needs, will not be served adequately. However, European health systems are mostly publicly funded and highly regulated; therefore, the unequal distribution is a consequence of wrong public regulation.

To measure the inequality between geographic areas, it is necessary to define what an appropriate geographic area is. A definition of geographic areas as units of analysis highly depends on the health resource the inequality of which is to be evaluated. Generally, geographic areas are smaller for an analysis of distribution of outpatient services, larger for an analysis of distribution of inpatient services, and very large for highly specialized services. Because national statistical offices usually collect data for administrative units, the areas that researchers analyze are states, provinces, regions, counties, and districts. But those administrative units do have to be related to hospital service areas.

Equal distribution of health resources is not only about equity but also about efficiency. Suppose that two regions, A and B, have populations with the same health needs, inhabitants of both regions pay taxes and health insurance, but the regions differ, for example, in the numbers of physicians per capita. Suppose that all physicians are under contract to the public health system and that there are more physicians in region A. However, one cannot see any reason why the public health system should finance the higher number of physicians in region A. Health needs being equal in both regions, there is apparently a relative oversupply of physicians in region A and a relative undersupply of physicians in region B. Assuming that marginal social benefit from health services provided by physicians are decreasing, an unequal allocation in the numbers of physicians is not efficient: patients from region A get lower marginal social benefits than patients in region B could possibly get in case that physicians would be reallocated from region A to region B. Hence, the total social benefit for the whole society is lower than it could be. In this situation, public regulation focused on equal distribution of physicians can improve the equity as well as the efficiency.

The objective of this study is to develop a measure of the geographic inequality in the case of multiple health resources. The proposed method is based on data envelopment analysis (DEA) and is simpler than the first version of the method described in Ref. (3). The proposed inequality measure is illustratively applied to the geographic inequalities in the distribution of health resources in the Czech Republic. The objective of this study also is to show that the models originally developed for measuring efficiency can be used to measure inequality.

## Literature Review

The existence of significant geographic differences in health resources, health expenditures, the utilization of health services, and health outcomes have been documented by a lot of studies from various countries of the world. For example, Johnston and Wilkinson (4) studied the distribution of general practitioners in Australia between 1986 and 1996. They used crude mortality as a measure of community need for medical services. The Robin Hood Index (RHI) was used as a measure of overall distribution. Nationally, the number of people sharing each general practitioner fell by 11% from 1,038 in 1986 to 921 in 1996. However, in 41 of 57 areas, the number of people per general practitioner actually increased over this period, indicating increasing inequity in the distribution. Over the decade, the number of relatively under-served areas increased from 67% in 1986 to 79% in 1996. Thus, despite the increasing number of general practitioners overall, the rural and remote parts of Australia became increasingly poorly served.

The OECD study (5) brings information about geographic variations in health-care utilization within and across 13 OECD member countries. The analysis focuses on a selected set of high-volume and high-cost health-care activities. Health-care utilization is recorded at the patient’s place of residence. Hence, the level of use in a given area cannot be explained by patients receiving treatment in other geographic areas. While the analysis in this study does not allow to determine precisely how much of these variations are unwarranted, some of these variations are too large to be explained solely by patient needs and/or preferences.

Dlouhý (6) analyzed the geographic distribution of doctors and hospital beds in 13 European countries (Austria, Bulgaria, Czech Republic, France, Hungary, Italy, Norway, Poland, Portugal, Romania, Slovakia, Spain, and Turkey). The RHI was calculated to measure regional inequalities in the distribution of doctors and hospital beds among the NUTS 2 regions. In 10 of 13 countries, the differences in regional distribution are higher for doctors than those for hospital beds. The highest regional inequalities in the case of doctors were found in Slovakia, Hungary, and Turkey. In the case of hospital beds, the highest inequalities were identified in Portugal, Spain, and Poland.

Most comparisons of health data in Europe take place at the national level. However, there is an increased interest in looking at health data at a sub-national level. This is, to some extent, because of the increased importance of regions in many countries. Regional information allows health professionals and decision makers to better understand the characteristics of their own region in wider context (7). While some regional variations reflect differences in patient needs and/or preferences, others do not. Instead, they are due to variations in medical practice styles, the ability of providers to generate demand beyond what is clinically necessary, or to unequal access to health-care services. These unwarranted variations raise concerns about the equity and the efficiency of health systems.

## Materials and Methods

### Inequality Measurement

Measures of inequality express the complexity of variation in observed variable by a single number. There is a variety of inequality measures described in the literature (8, 9), and one cannot say that one measure is clearly better than the other. The simple measures of inequality are the ranges, which use only data on the extreme values. The absolute range is defined as a difference between the maximum and minimum observed values per capita. The relative range is defined as the absolute range divided by the average number of units per capita for the entire population. Other measures of this type are decile ratios. By concentrating on the geographical areas with extreme or selected values only, all these indices give a limited view on the overall distribution.

The most popular measure of inequality that uses all observations is the Gini coefficient. The Gini coefficient is derived from the Lorenz curve, a cumulative frequency curve that compares the empirical distribution of the studied variable with the uniform (egalitarian) distribution that represents perfect equality. The Gini coefficient ranges between 0, which occurs in the case of perfect equality, and 1, which occurs in the case of perfect inequality. The RHI (also known as the Pietra ratio) measures what proportion of resources has to be moved from areas with above-average provision to areas with below-average provision to achieve equal distribution. Graphically, the RHI represents the maximum vertical distance from the Lorenz curve to 45° egalitarian line. In our view, the advantage of the RHI over the Gini coefficient and other inequality measures, such as the Atkinson index, coefficient of variation, and the generalized entropy measure, is its clear practical interpretation. The RHI is calculated by the formula:

$$\mathrm{RHI}=\frac{1}{2}\sum_{i=1}^{n}|\pi_{i}-\rho_{i}|,$$

where π*_i_* is the population proportion, ρ*_i_* is the resource proportion, and *n* is the number of geographic areas. The index is multiplied by 100 to be in percentages.

### Data Envelopment Analysis

Data envelopment analysis was developed to construct the production frontier and evaluate the technical efficiency of production units. DEA is a method based on the theory of mathematical programming that estimates the production frontier as the piecewise linear envelopment of the observed data. The first DEA model for multiple inputs and outputs was formulated in 1978 (10). Consequently, a variety of DEA models with many extensions and modifications has been developed. These extensions can be found in textbooks that also present many examples of applications from both private and public sectors (11–13), including health care (14–16). For example, Hollingsworth (15) reviewed 188 published papers on the frontier efficiency measurement in health care. DEA alone was used in 50% of studies; a quarter of studies used regression analysis in two stage analysis, typically to regress factors on the efficiency scores in an attempt to determine influences on efficiency. Stochastic frontier analysis and other parametric frontier techniques were used in 12% of studies; Malmquist techniques were used in 9% of studies. O’Neill et al. (16) reviewed 79 DEA hospital efficiency studies published from 1984 to 2004 that represent 12 countries. Their cross-national comparison reveals significant differences with respect to important study characteristics such as type of DEA model selected and choice of input and output categories. Compared with the U.S. studies, European efforts are more likely to measure allocative rather than technical efficiency, use longitudinal data, and use fewer observations.

The production unit uses a number of inputs to produce outputs. The relative technical efficiency of the production unit is defined as the ratio of its total weighted output to its total weighted input or, *vice versa*, as the ratio of its total weighted input to its total weighted output. DEA allows each production unit to choose its own weights of inputs and outputs in order to maximize its efficiency score. A technically efficient production unit is able to find such weights that it lies on the production frontier. The production frontier represents the maximum amounts of output that can be produced by given amounts of input (in the output maximization model) or, alternatively, the minimum amounts of inputs required to produce the given amount of output (in the input minimization model).

The DEA model calculates for each production unit efficiency score, the relative weights of inputs and outputs. The model also identifies peers for each production unit that is not technically efficient. The peers of a technically inefficient production unit are technically efficient production units with similar combinations of inputs and outputs that serve as benchmarks showing potential improvements that the unit can attain. Because the peers are real production units, one can expect that the efficiency improvements should be attainable by the inefficient units.

**Figure 1:**
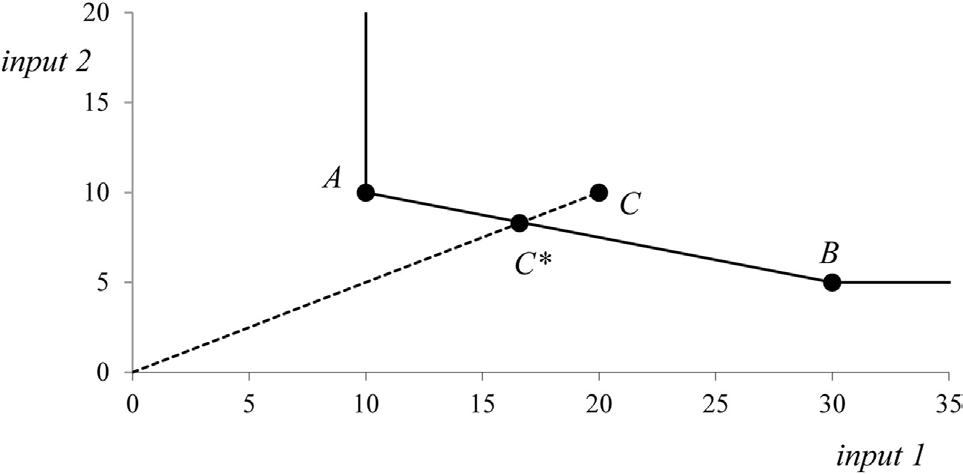
Data envelopment analysis production frontier with 2 inputs and 1 output.

A construction of a production frontier and calculation of efficiency scores by the constant returns-to-scale DEA model are shown in Figure 1. Let us suppose that there are three production units A, B, and C. These production units produce the same level of single output with two inputs A = (10, 10), B = (30, 5), and C = (20, 10). The production units A and B are technically efficient, and they both lie on the production frontier. The production unit C uses more inputs than it is technically necessary; therefore, this unit lies above the production frontier and is technically inefficient. The units A and B are the peers showing to unit C how to reduce both inputs to be technically efficient. The hypothetical production unit C* is a linear combination of real units A and B. The hypothetical production unit C* represents the efficient alternative in which all inputs of original unit C are reduced proportionately. However, it should be noticed that unit C can achieve technical efficiency by moving to any position on the production frontier.

Suppose we have *n* production units that use *m* inputs to produce *r* outputs. The mathematical formulation of the input-oriented version of the constant returns-to-scale DEA model for production unit *q* is:

$$\text{Maximize }\varphi_{q}=\sum_{k=1}^{r}u_{k}y_{kq},$$

$$\text{subject to }\sum_{k=1}^{r}u_{k}y_{\mathrm{kj}}-\sum_{i=1}^{m}v_{i}x_{\mathrm{ij}}\leq \text{0 , }j=\text{1, 2},\ldots ,n\text{,}$$

$$\sum_{i=1}^{m}v_{i}x_{\mathrm{iq}}=1,$$

$$u_{k}\geq \varepsilon ,\;i=1,2,\ldots ,r,$$

$$v_{i}\geq \varepsilon ,\;j=1,2,\ldots ,m.$$

where φ*_q_* is the technical efficiency score, *x_ij_* is the amount of input *i* used by production unit *j, y_kj_* is the amount of output *k* produced by production unit *j*, and ε is an infinitesimal constant. The output weights *u_i_* and input weights *v_j_* are variables in the DEA model. In the input-oriented model, the efficiency score φ*_q_* is one if the unit *q* is technically efficient, and is lower than one if the production unit is technically inefficient. The efficiency score measures a size of input reduction that makes production unit *q* technically efficient. In the output-oriented model, the efficiency score is one if the unit *q* is technically efficient and is greater than one if the unit is technically inefficient. To obtain the technical efficiency scores for all production units, the DEA model has to be solved for each production unit.

### The Multiple Resource Inequality Measure

Suppose that we should measure inequality in geographic distribution in the case of multiple health resources. We can do that by using traditional measures described in the Inequality Measurement section separately for each health resource, but by doing that, we miss the possibility of substitution between health resources. For example, the health resources as physicians and nurses are, at least to some extent, substitutes. Hence, the region with fewer physicians may compensate such disadvantage by a larger number of nurses. In such a case, the total inequality is lower than expected from separate measurement. To cope with cases with multiple health resources, one can use multiple criteria decision-making for setting relative resource weights. Such weights are then used nationwide for all regions. The question is how to obtain such weights and if such assumption about resource substitution is right. More flexible approaches to estimate resource weights and the rate of substitution are based on the production function. Health resources are inputs and population (as a measure of health need) is the single output. The production function can be estimated by econometric methods or by DEA on which we will focus on.

Let us assume a situation with two resources (inputs) and one output that can be illustrated by Figure 1. Production units A, B, and C now represent regions and the output is the regional population that serves as an estimation of health need. Regions A and B that lie on the production frontier are technically efficient, and region C is inefficient, for example, with the input-oriented efficiency score φ*_3_* = 0.8. A lower level of inefficiency in this situation represents a higher level of resources that are available for regional population. The efficiency score of the input-oriented constant returns-to-scale model, which is lower than one, expresses the excess of resources above the most badly served regions that are represented by the set of DEA efficient units. Thus, it is good to live in inefficient regions! The efficiency score 0.8 means that there is a possibility of 20% resource reduction in the given region or that the given combination of resources is able to serve a 25% larger population (1/0.8). By the efficiency scores for regions, multiple health resources are now transformed into a single virtual resource the amount of which is calculated as a regional population (or a proportion of regional population) multiplied by reciprocal value of the efficiency ratio φ*_i_*.

The following method of inequality measurement is proposed:

1. For each region, calculate the efficiency score by the input-oriented version of the constant returns-to-scale DEA model with health resources as inputs and the regional population (or other measure of health need) as output.
2. Calculate the value of virtual health resource ρ_i_* = π*_i_*/φ*_i_*.
3. Calculate the RHI for the virtual health resource.

Although the value of the RHI cannot be directly interpreted as in the case of original health resources, it combines all health resources in one dimension. If all regions are technically efficient by the DEA model, then the RHI is 0. Suppose that there exist only regions A and B on Figure 1. Due to possibility of resource substitution, both regions are technically efficient and the RHI will be 0. However, the separate RHIes for input 1 and input 2 will return positive values, so some level of reallocation will be needed. On the other hand, the maximum theoretical value of the RHI is 1. Note that a virtual resource can also be interpreted as a virtual population in the constant returns-to-scale DEA model. The inequality measure can be thus interpreted as the percentage of the population that has to move from more technically efficient regions to less technically efficient regions to achieve technical efficiency (i.e., geographic equality).

### Data

The proposed inequality measure is applied to the regional data from the Czech Republic. The Czech Republic is a Central-European country with 10.5 million inhabitants. The country is divided into 14 regions with populations ranging from 298,506 inhabitants (Karlovarsky region) to 1,320,721 inhabitants (Stredocesky region). The data of two regions, Prague and Stredocesky regions, were joined together because the Prague region, the capital, is located inside the territory of the Stredocesky region. We assume that the population of the Stredocesky region use frequently health services in the capital. In the Czech Republic, the health services are paid by public health insurance, which should guarantee equal access to services for the whole population. Equal distribution of physicians and nurses is thus an important health policy issue. All data come from the year 2015 and were obtained from the Institute of Health Information and Statistics of the Czech Republic (17). In this study, we consider two inputs: the number physicians in full-time equivalents and the number of nurses in full-time equivalents. The output is the regional population. In some inequality studies (4), the crude death rate was used as a measure of health need by adjusting the number of population. It is assumed that the crude death rate will be higher in older populations than in younger populations.

## Results

The regional characteristics of 13 Czech regions are presented in Table 1. In this study, we consider two health resources: physicians and nurses. The number of physicians per 10,000 inhabitants ranges from 28.98 to 43.50, with the national average being 36.30. The number of nurses per 10,000 inhabitants ranges from 63.20 to 83.83, with the national average being 77.48. We assume that substitution between physicians and nurses is possible. In a region with more physicians, the intensity of care is higher, so, a lower number of nurses is needed, and *vice versa*, in a region with more nurses, a lower number of physicians is needed.

**Table 1:** Health resources, efficiency, and virtual resource by region, Czech Republic, 2015.

Region	Population	Number of physicians	Number of nurses	Efficiency	Virtual resource
Prague + Stredocesky	2,583,228	11,237	21,654	0.754	3,426,536
Jihocesky	637,292	2,030	4,288	0.943	675,779
Plzensky	575,665	2,181	4,575	0.795	723,897
Karlovarsky	298,506	1,019	2,337	0.860	347,233
Ustecky	823,381	2,386	5,713	1.000	823,381
Liberecky	439,152	1,321	2,775	1.000	439,152
Kralovehradecky	551,270	1,948	4,424	0.833	661,880
Pardubicky	516,247	1,523	3,449	0.998	517,049
Vysocina	509,507	1,566	3,945	0.943	540,448
Jihomoravsky	1,173,563	4,695	9,809	0.756	1,552,184
Olomoucky	635,094	2,458	5,505	0.764	831,586
Zlinsky	584,828	1,729	4,011	0.990	591,015
Moravskoslezsky	1,215,209	4,172	9,203	0.865	1,405,613
Czech Republic	10,542,942	38,268	81,688	*x*	12,535,755

The values of separate RHIes are 6.64% for physicians and 3.96% for nurses. Thus, more than 6% of Czech physicians should be reallocated between the regions. The situation in the case of nurses is better than for physicians and, in fact, it is not so far from equal distribution. In the next step, we use combined RHI for both health resources. First, the technical efficiency ratios were obtained by the input-oriented constant returns-to-scale DEA model with two inputs (the number physicians and the number of nurses) and one output (the regional population). The efficiency ratios are presented in Table 1. Second, the values of virtual health resource for each region were then calculated (Table 1). Third, the RHI was applied to virtual health resource. Its value 5.06% takes into account that the combinations of two health resources serve regional populations.

## Discussion

The inequality measure that is able to deal with multiple health resources by transforming them into a single virtual resource was formulated on the basis of the DEA model. Insofar as to the best knowledge of the author, the inequality measure presented in this paper is novel to both the inequality measurement and the DEA literature. The study shows that the DEA originally developed for measuring efficiency can be used successfully to measure inequality.

For the illustrative purpose, the proposed inequality measure was calculated for the Czech Republic. The values of separate RHIes are 6.64% for physicians and 3.96% for nurses. The combined RHI was 5.06%. It is evident that the value of the RHI will never be 0 in the reality and the Czech values are not so far from equal distribution. The health system of the Czech Republic is performing relatively well in the international comparison (6).

The important methodological issue is the measurement of inequality in cases of metropolitan regions surrounded by other regions. In this study, the data of two regions were joined.

## Conclusion

The existence of geographic differences in the distribution of health resources is a reality in both developing and developed countries. So, the inequality measurement is an important methodological tool that helps policy makers and researchers in evaluating the degree of inequality. The inequality measure that is able to deal with multiple health resources by transforming them into a single virtual resource was formulated on the basis of the DEA model. The proposed inequality measure was applied to the Czech Republic for calculating inequalities in the distribution of physicians and nurses.

There are two possible directions of future research: first, the use of other DEA models for inequality measurement; second, formulations of the DEA-based versions of other inequality measures. The issue, which is not addressed here is that all inequality measures implicitly assume that historically developed average levels of health resources are the right ones.

## Author Contributions

The author is the sole author of the work described in this manuscript. The author planned the study, analyzed the data, and wrote the article.

## Conflict of Interest Statement

The author declares that the research was conducted in the absence of any commercial or financial relationships that could be construed as a potential conflict of interest.
